# Toward a real time multi-tissue Adaptive Physics-Based Non-Rigid Registration framework for brain tumor resection

**DOI:** 10.3389/fninf.2014.00011

**Published:** 2014-02-17

**Authors:** Fotis Drakopoulos, Panagiotis Foteinos, Yixun Liu, Nikos P. Chrisochoides

**Affiliations:** ^1^CRTC Lab and Computer Science, Old Dominion UniversityNorfolk, VA, USA; ^2^Computer Science, College of William and MaryWilliamsburg, VA, USA; ^3^Radiology and Imaging Science, National Institutes of HealthBethesda, MD, USA

**Keywords:** non-rigid registration, tumor resection, finite element method, biomechanical model, ITK, real time

## Abstract

This paper presents an adaptive non-rigid registration method for aligning pre-operative MRI with intra-operative MRI (iMRI) to compensate for brain deformation during brain tumor resection. This method extends a successful existing Physics-Based Non-Rigid Registration (PBNRR) technique implemented in ITKv4.5. The new method relies on a parallel adaptive heterogeneous biomechanical Finite Element (FE) model for tissue/tumor removal depicted in the iMRI. In contrast the existing PBNRR in ITK relies on homogeneous static FE model designed for brain shift only (i.e., it is not designed to handle brain tumor resection). As a result, the new method (1) accurately captures the intra-operative deformations associated with the tissue removal due to tumor resection and (2) reduces the end-to-end execution time to within the time constraints imposed by the neurosurgical procedure. The evaluation of the new method is based on 14 clinical cases with: (i) brain shift only (seven cases), (ii) partial tumor resection (two cases), and (iii) complete tumor resection (five cases). The new adaptive method can reduce the alignment error up to seven and five times compared to a rigid and ITK's PBNRR registration methods, respectively. On average, the alignment error of the new method is reduced by 9.23 and 5.63 mm compared to the alignment error from the rigid and PBNRR method implemented in ITK. Moreover, the total execution time for all the case studies is about 1 min or less in a Linux Dell workstation with 12 Intel Xeon 3.47 GHz CPU cores and 96 GB of RAM.

## Introduction

The Non-Rigid Registration (NRR) between pre-operative (pre-op) MRI data and the *in-situ* shape of the brain (iMRI) can compensate for brain deformation during the Image-Guided Neurosurgery (IGNS). The requirements for NRR are: *accuracy and speed*. In this paper, we focus on both aspects and propose a framework to address one of the two important challenges of NRR for IGNS: brain deformation due to tumor resection. The other challenge is tissue retraction (Miga et al., 2001; Platenik et al., 2002), which is outside of the scope of this paper.

Modeling the behavior of the brain remains a key issue in providing navigation for IGNS. Warfield et al. (2000) proposed a two step NRR method that accurately simulates the biomechanical properties of the brain and its deformations during surgery. In the first step, an active surface algorithm iteratively deforms the surface of the first brain volume to match that of the second volume (Ferrant et al., 1999). In the second step, the volumetric brain deformation implied by the surface changes is computed in parallel via a biomechanical model. In our work, we estimate the correspondences for brain volume data instead of brain surface data. Additionally, the selection of the image features, the computation of the correspondences, and the volumetric brain deformations are all performed in parallel.

Mostayed et al. (2013) presented a biomechanical-based registration method which does not require an intra-operative (intra-op) MRI to update the pre-op MRI, but only some sparse intra-op data points. The method was compared to a BSpline algorithm (Rueckert et al., 1999) and was qualitatively and quantitatively evaluated in 13 clinical cases. In our work, we use approximation methods instead of interpolation techniques, which are sensitive to outliers because of noise in the iMRI.

Clatz et al. (2005) presented a robust volumetric NRR static scheme to capture brain shift from intra-op MRI. This method was validated with retrospective data and later Archip et al. (2007) published a prospective study on NRR of pre-op imaging (T1, fMRI, DTI) with intra-op images (T1). These clinical studies used iMRI data obtained from 11 patients enrolled over 12 months. Chrisochoides et al. (2006) parallelized some of this method's computational intensive components and used a cluster of 300 workstations to calculate the image alignments in near real-time. In our work, we extend this work in several directions: (1) in near real-time we adaptively change the FE model in order to improve the quality of elements and handle larger deformations, (2) we use a multi-tissue mesh generation to incorporate a more accurate heterogeneous FE model, (3) we improve upon the parallelization techniques and achieve better performance compared to the earlier code, and (4) rely only on ITK rather than on a proprietary library.

All of the above methods and others, such as Rexilius et al. (2001) and Ferrant et al. (2001), compensate only for the brain shifts (BS) occurring during a craniotomy. The shifts mainly occur from the cerebro spinal fluid (CSF) leakage, the impact of the gravity on the brain tissue, the edema, and the administration of osmotic diuretics. They do not address tumor resection, which invalidates the biomechanical model defined on the pre-op MRI. In this paper, we propose a method to address this challenging problem; however, some deep brain tumor resection cases that our method is not able to handle within the accuracy we would like to achieve. We have an ongoing project to address deep brain tumor resection cases.

Similar to 3D adaptive mesh generation efforts we present here, but for 2D only, Risholm et al. (2009) presented an adaptive FE multi-level grid registration scheme that accommodates a superficial tumor resection. The method was evaluated in 2-dimensioanl medical and synthetic images. The method consists of creating a planar FEM grid, then checking each element to determine whether the similarity metric per unit area over the element or a set of elements area is greater than a threshold. Whenever both of these conditions are true, a Delaunay split strategy is applied. In our work, we do not split the elements, but we generate a new global 3D Delaunay mesh of high quality tetrahedral elements in parallel. We show that the new generated mesh captures not only the small brain deformations, but also the complex geometric changes around the tumor cavity.

Periaswamy and Farid (2006) developed a robust Expectation-Maximization (EM) framework for the simultaneous segmentation and registration of a pair of 3D clinical images with partial or missing data. A MatLab implementation of this method required 30 min to register a pair of 64 × 64 × 64 volumes on a 2.8 GHz Linux machine. In our work, we introduce several parallel components, thus, we can register adult brain MRIs with resolution 250 × 219 × 176 in almost 1 min. Specifically, the end-to-end execution time in our shared memory workstation with 12 Intel Xeon 3.47 GHz CPU cores and 96 GB of RAM, is between 34 and 60 s.

Finally, in our previous work (Liu et al., 2010), we developed a nested EM registration framework to take into account the tumor resection by iteratively rejecting feature outliers and element outliers. In this study, instead of an explicit rejection of tumor elements, we generate a new multi-tissue tetrahedral mesh which adapts to a gradually warped segmented image.

In summary, this paper augments the ITK software implementation in Liu et al. (2014) and proposes an Adaptive Physics-Based Non-Rigid Registration (APBNRR) framework, compensating for brain deformations induced by a tumor resection. The contributions of this paper are:

- The design and implementation of a new framework to develop a parallel adaptive heterogeneous biomechanical FE model that will accurate compute brain deformations required for the NRR of MRI data with tumor resection.
- The near real-time performance (about 60 s) of the framework for NRR of volume brain MRIs.

We evaluate this framework qualitatively and quantitatively with 3D clinical MRI data, including BS from Archip et al. (2007), as well as both partial and complete tumor resections (CTR). For the qualitative evaluation, we employ image subtractions; for the quantitative evaluation, we use the Hausdorff distance (HD) metric. Our evaluation on 14 patients indicates that our scheme is reducing the alignment error up to 6.61 and 4.95 times compared to a rigid and the publicly available NRR method PBNRR (Liu et al., 2014) of ITKv4.5, respectively. There is a statistically significant difference between the accuracy of the alignment of pre- and intra-operative images, with and without NRR. Compared to rigid registration and non-rigid PBNRR method, our method demonstrates significant improvement with *P*-values 2.673E-06 and 4.533E-05, respectively, (significant level 0.05). The detailed results are presented in Table 4.

In the next section we will describe the proposed framework and introduce the new modules to manage and integrate the FE model adaptivity in almost real-time.

## Materials and methods

### Patient population

In our study, we evaluated our new method on total 14 patients: a group of 10 patients (6 female, 4 male; age range: 28–62 years; mean: 45.2 year with supratentorial gliomas) and a group of four patients (2 female, 2 male; unknown age but withdrawn form a pool of 24 patients with an age range of 17–70 years). The 10 patients from Brigham and Women's Hospital in Boston underwent surgery using the intraoperative MR image–guided therapy facility between April 2005 and January 2006 for tumors in and adjacent to eloquent brain areas (such as the precentral gyrus and cortico spinal tract, for motor function; and Broca's and Wernicke's areas, for language function). The remaining four patients underwent surgery at Huashan Hospital, Fudan University in Shanghai, China from September 2010 to August 2013. The MRI data of the 10 cases from BWH were acquired with the protocol: whole brain sagittal 3D-SPGR (slice thickness 1.3 mm, *TE*/*TR* = 6/35 ms, *FA* = 75°, FOV = 24 cm, matrix = 256 × 256). For the four cases of Huashan Hospital, the MRI data were acquired (IMRISneuro, IMRIS, Canada) in 8 min with the protocol: 3D T1-weighted magnetization-prepared rapid gradient echo (MPRAGE) sagittal images with [dimension = 256 × 256 × 176, in plane resolution = 1.0 × 1.0 mm, thickness = 1.0 mm, FOV = 256 × 256]. Both data collections were carried out with Institutional Review Board (IRB) approval from both Hospitals.

### Non-rigid registration

**Figure 1:**
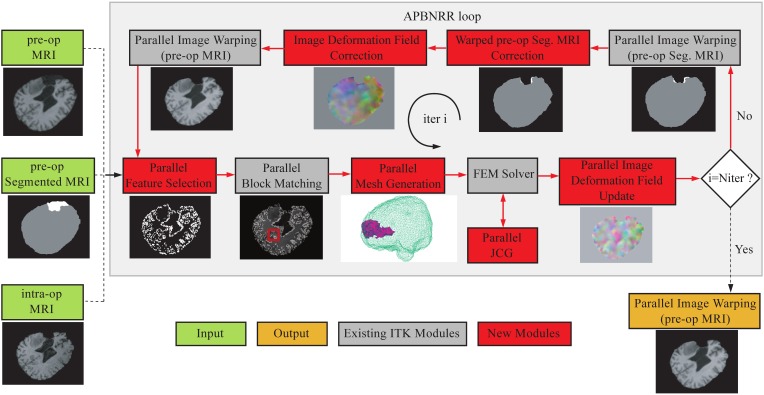
**The APBNRR framework**. The red boxes represent the new contributions and the gray boxes the existing ITK modules. The red arrows show the execution order of the different modules. The APBNRR loop breaks when the desired number of iterations has reached. The output image (orange box) is the warped pre-op MRI when *i* = *N*_iter_.

The APBNRR framework is built on top of the ITK open source system. Figure 1 illustrates the modules of the framework. The red and gray boxes depict the new and existing ITK modules, respectively. The new parallel modules are based on POSIX thread library.

In Figure 1, the green boxes indicate the input, consisting of a pre-op MRI, an intra-op MRI, and a two-tissue segmentation (brain parenchyma, tumor) of the pre-op MRI. The orange box represents the output, a warped pre-op MRI. Prior to APBNRR, three pre-processing steps are applied: (1) the extraction of the brain from the skull with the BET tool (Smith, 2002), (2) the segmentation of the brain parenchyma and the tumor in the pre-op MRI, and (3) the rigid alignment of the pre- and the intra-op MRI with the 3D Slicer^1^. Consequently, the green boxes in Figure 1 represent the input after these three steps. The tumor and the brain parenchyma in the segmented images are shown with white and gray, respectively. The red arrows represent the execution order of the modules. As shown in Figure 1, the input pre- and pre-op segmented MRI are iteratively warped toward the intra-op MRI. During the first iteration (*i* = 1), the warped images are set equal to the corresponding input images. In the rest of this section, we briefly describe all APBNRR modules presented in Figure 1.

First, we define the existing ITK modules for completeness. Detailed descriptions for the existing ITK modules are given in Liu et al. (2014) and in the ITK API documentation^2^. The Parallel Feature Selection is a multi-threaded implementation of ITK's sequential method and selects highly discriminant features (blocks of voxels) from the warped pre-op MRI. Parallel Block Matching (Liu et al., 2014) computes the displacements between the selected feature blocks and blocks in the intra-op MRI. The Parallel Mesh Generation (Foteinos and Chrisochoides, 2013) creates a two-tissue tetrahedral mesh from the warped pre-op segmented MRI. The FEM Solver (Clatz et al., 2005; Liu et al., 2014) builds a FE biomechanical model, applies the block matching displacements to the model, and estimates the mesh deformations iteratively by first using an approximation method. Once many of the outliers from block matching are rejected, we use an interpolation formulation to compute the deformation field. The Parallel Jacobi Conjugate Gradient (JCG) is a multi-threaded implementation of the sequential JCG in ITPACK (Grimes et al., 1978) for solving sparse linear systems of equations. The Parallel Image Deformation Field Update transforms the computed mesh deformations to an image deformation field and adds the previous (i − 1, i − 2, … 1) image fields to the current (ith) image field.

The loop in Figure 1 breaks when the desired number of iterations has reached (*i* = *N*_*iter*_). In this case, the Parallel Image Warping module creates the output warped pre-op MRI with the updated image deformation field. *N*_iter_ is the number of adaptive iterations specified by the user (Table 3). According to our experimental evaluation, a satisfactory registration accuracy can be achieved when 3 ≤ *N*_iter_ ≤ 5. In the case where *i* ≠ *N*_iter_, the pre-op segmentation is automatically warped with the updated image field. Afterwards, the warped pre-op segmentation and the updated field are corrected to take into account the resected tissue, by the module's Warped pre-op Segmented MRI correction and Image Deformation Field Correction, respectively, (Figure 1). Subsequently, the warped pre-op MRI is generated with the corrected deformation field. In the next loop, the feature blocks are selected from the latest warped pre-op MRI, and the mesh is generated from the latest corrected warped segmented pre-op MRI.

In the remaining subsections, we describe in more detail the new contributed modules.

### Parallel feature selection

The Parallel Feature Selection is a multi-threaded implementation of ITK's sequential method. The input of the module is an image and a mask: as an input image we use the warped pre-op MRI; as an input mask we use the corrected warped pre-op segmented MRI. The output of the module is a set of highly discriminant features in the input image. Each feature is a block of voxels restricted inside the mask. Parameter *F_s_* (Table 3) determines the percentage of the selected blocks from the total number of blocks. Parameter *B*_sx_ × *B*_sy_ × *B*_sz_ (Table 3) determines the size of the block in x, y, z (axial, coronal, sagittal) image directions. More details about the feature selection parameters can be found in Liu et al. (2014).

Initially, the parallel module partitions the input image into *k* sub-regions, where *k* is the number of threads. Each thread computes a variance value and an image index for each feature inside the sub-region. Then, all the computed pairs are sorted in parallel according their variance and merged into a global vector. At this point, the size of the global vector equals the total number of image feature blocks. Next, the module selects ⌊0.5 + *N*_Features_ × *F_s_*⌋ blocks from the global vector in parallel.

Because the selection is performed in parallel, the feature blocks are picked from the global vector in a different order compared to the sequential method. A prohibited connectivity is used according to Liu et al. (2014) to avoid overlap between the selected blocks, which means that after a feature is selected, it's connected features are no longer candidates for selection. Therefore, the output feature blocks of the sequential and parallel implementations are generally different. In our module, we first partition the global vector into sub-vectors, and then k threads simultaneously select the features from each sub-vector. The number of partitions can be adjusted with the parameter *N*_Part_. The larger the *N*_Part_, the smaller the size of each sub-vector and the greater similarity between the selected features of the parallel and the sequential implementation; however, very large values for *N*_Part_ increase the execution time of the module because of the more frequent thread synchronizations.

For an average 3D brain MRI with about 10^6^ total feature blocks, the experiments have shown that a good balance between the speed of the module and the similarity between the sequential and the parallel selected features can be achieved when 100 ≤ *N*_Part_ ≤ 2.0. In the conducted experiments of this paper we used *N*_Part_ = 100. The parallel module exits when ⌊0.5 + *N*_Features_ × *F_s_*⌋ feature blocks are selected.

### Parallel mesh generation

The parallel mesh generation module is based on the Delaunay meshing and refinement algorithm developed in our group by Foteinos and Chrisochoides (2013). Let us denote the mesh in the beginning of an APBNRR iteration *i* with *M_i_*. Initially, *M*_1_ is generated for the pre-op segmented MRI. After the completion of iteration *i*, the displacements are computed on the nodes of *M_i_*, which yields the deformed mesh *M_i_*'. *M_i_*' is not used for the next loop because it consists of distorted elements of poor quality in terms of dihedral angles and aspect ratio. Instead, the warped pre-op segmented MRI is used in the next iteration as the input of the mesher, which produces in parallel a new global mesh *M*_*i* + 1_ of high quality elements. Parameter δ (Table 3) determines the size of the mesh (δ > 0). The smaller the δ, the larger the mesh.

Table 1 depicts the minimum and maximum dihedral angles α, before and after the Parallel Mesh Generation module for an example with *N*_iter_ = 4 and δ = 5. At iteration *i*, the “before” corresponds to the deformed mesh at *i* − 1 and the “after” to the new generated mesh at *i*. The #Tets refers to the number of tetrahedral elements in the new generated mesh at *i*. As shown in Table 1, after the mesh generation, the α_min_ increases and the α_max_ decreases, thus the poor quality elements are eliminated (e.g., the larger the min dihedral angles and smaller the max dihedral angles of the elements, the faster and better the condition number and thus, the convergence of the linear solver).

**Table 1:** **The minimum and maximum dihedral angles α, before and after the Parallel Mesh Generation (*N*_iter_ = 4, δ = 5)**.

**Iteration *i***	**#Tets**	**α_min_**		**α_max_**	
		**Before**	**After**	**Before**	**After**
1	14,278	–	5.00°	–	169.68°
2	13,482	0.34°	5.24°	179.28°	169.92°
3	13,497	0.14°	4.91°	179.79°	169.71°
4	12,957	0.10°	5.01°	179.80°	171.32°

### Parallel JCG

The JCG method is an adaptive accelerated semi-iterative algorithm for solving linear systems A·x = b (1), where A in *R^N × N^* symmetric or nearly symmetric positive definite matrix and b, × b in *R^N^*a given column and solution, respectively. *N* = 3*n* where *n* is the number of mesh vertices, A is the stiffness matrix of the biomechanical model, b is the block matching displacement vector, and x is the mesh displacement vector. Grimes et al. (1978) presented the sequential JCG method, one of the seven ITPACK subroutines used in ITK to solve linear systems like (1). In this paper, we developed a new parallel implementation of the sequential JCG method. In our implementation, we use the original ITPACK matrix storage scheme, and we perform all the vector-vector and matrix-vector multiplications in parallel. There are other highly optimized linear solvers like the PETSc (Balay et al., 2000); however, for portability reasons, we decided (with Kitware Inc.) that they are not suitable for the ITK since their integration with ITK is cumbersome. In addition, ITPACK is part of ITK's distribution, so the integration of the new parallel JCG module is straight-forward. In our framework (Figure 1), the FEM Solver calls the Parallel JCG for the solution of system (1). The total number of JCG calls equals to *N*_iter_ × (*N*_appr_ + *N*_int_), where the parameters *N*_iter_, *N*_appr_, and *N*_int_ are given in Table 3. The FEM solver formulates the system (1) and estimates the mesh deformations (in our work with the Parallel JCG) from an approximation to an interpolation-based formulation while rejecting the outlier blocks (features with large error between the computed mesh deformations and the block matching displacement). Clatz et al. (2005) and Liu et al. (2014) give more details about the FEM Solver module (i.e., the method and its current ITK implementation, respectively).

### Parallel image deformation field update

This module takes as an input the estimated mesh deformations from the FEM Solver and produces as an output an image deformation field. The output field is additive. It holds the sum of the previous image fields (iterations 1, 2, …, i – 1) and the current image field (iteration *i*). The computations are accomplished in two steps: first, the module creates an image deformation field DF*_i_* from the mesh deformations; second, the DF*_i_* is added to the additive deformation field DF_addi_ − 1 of the previous iteration. The relation between DF_addi_ and DF*_i_* is given by the following Equation (2): DF_addi_ = DF_addi_ − 1 + DF*_i_*, *i* ≥ 1, where i is an APBNRR iteration, and DF_add0_ = 0. In order to avoid the interpolation of the warped images, the DF_addi_ is used for the image warping. Consequently, only the input images (pre-op and the input pre-op segmented MRI) are interpolated. The computation of the DF*_i_* and the addition at (2) are implemented in parallel. For the first step, the deformed mesh is partitioned into *k* sub-meshes, where *k* is the number of threads. Then each thread transforms the sub-mesh deformations into image deformations. For the second step, the image fields DF_addi_ − 1 and DF*_i_* are partitioned into *k* subfields, and the addition at (2) is performed in parallel.

### Warped pre-op segmented MRI correction

This module is the first of the two correction modules that take into account the resected tissue. The input consists of a warped pre-op segmented MRI and a brain mask of the intra-op MRI. BET (Smith, 2002) or 3D Slicer can easily create the brain mask. The output of the module is a corrected warped pre-op segmented MRI.

**Figure 2:**
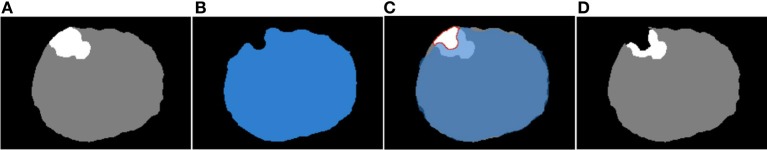
**The correction of the warped pre-op segmented MRI. (A) Input warped pre-op segmented MRI. (B)** Input intra-op mask MRI. **(C)** The **(A)** and **(B)** overlapped. **(D)** Output corrected warped pre-op segmented MRI at ith iteration.

The module overlaps the input images and detects the region of the tumor that falls into the mask background. Next, the voxel values of the detected region are set equal to zero. Figure 2 illustrates an example of this process where the same representative slice is shown in each image. Figures 2A,B show the input module images, Figure 2C shows the input images overlapped, and Figure 2D shows the output module image. The black, white, gray, and blue colors in the images correspond to the background, the tumor, the brain parenchyma, and the mask, respectively. From all the possible overlapped regions in Figure 2C, we target the portion of the white region that falls outside the blue region, or what we call the corrected region. The corrected region is delineated in Figure 2C with red and is comprised of white (tumor) voxels from Figure 2A and black (background) voxels from Figure 2B. Consequently, the voxels of Figure 2A that correspond to the corrected region of Figure 2C are modified as follows: white → black. The output corrected segmented image is shown in Figure 2D where a portion of the white (tumor) voxels of Figure 2A is replaced by black (background) voxels.

### Image deformation field correction

**Figure 3:**
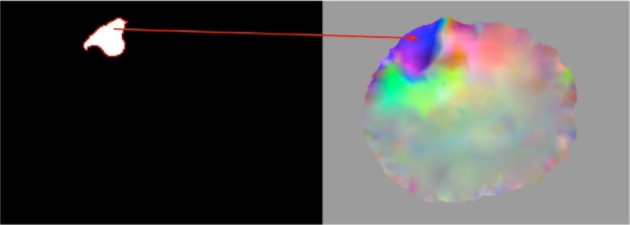
**Representation of the map between the corrected region of the warped pre-op segmented MRI (left) and the image deformation field DF_addi_ (right)**.

This module takes as an input the corrected region from warped pre-op segmented MRI (Figure 3, left) and the image deformation field DF_addi_ (see subsection Parallel JCG) depicted in Figure 3. Similar to the previous correction module, it detects the voxels in the DF_addi_ that correspond to the input corrected region and sets their deformation values equal to zero. The detection of the voxels in the DF_addi_ is accomplished by mapping the input corrected region to the DF_addi_. Figure 3 demonstrates the map with a red arrow. In other words, the module makes the DF_addi_ consistent with the corrected segmented MRI described in subsection Warped pre-op Segmented MRI Correction.

## Results

We evaluate our framework on 14 clinical 3D MRI cases (8 females and 6 males patients). All data are anonymized and an IRB is granted. The Surgical Planning Laboratory at Brigham and Women's Hospital (Talos and Archip, 2007) provided 10 cases, and the Department of Neurosurgery at Shanghai Huashan Hospital (Chen et al., 2008) provided four cases. Depending on the type of tumor resection depicted in the intra-op MRI (i.e., just brain shift but no tumor resection, or partially/completed resected), the cases are categorized as BS, Partial Tumor Resections (PTR), and CTR, respectively. From a total of 14 cases, 7 are BS, 2 are PTR, and 5 are CTR. Table 2 lists the provided clinical data.

**Table 2:** **The clinical MRI data of this study**.

**Case**	**Type**	**Provider**	**Genre**	**Tumor location**	**Histopathology**
1	BS	B&W	M	L perisylvian	Oligoastrocytoma WHO II/IV
2	BS	B&W	F	R occipital	Anaplastic Oligodendroglioma WHO III/IV
3	BS	B&W	M	R frontal	Oligoastrocytoma WHO II/IV
4	BS	B&W	F	L posterior temporal	Glioblastoma
5	BS	B&W	F	L frontal	Oligodendroglioma WHO II/IV
6	BS	B&W	M	R frontal	Oligodendroglioma WHO II/IV
7	BS	B&W	F	R occipital	n/a
8	PTR	B&W	F	L frontal	Glioblastoma multiforme (WHO IV/IV)
9	PTR	Huashan	M	L frontal	Glioblastoma multiforme (WHO IV)
10	CTR	B&W	M	Fronto-temporal	Oligodendroglioma WHO II/IV
11	CTR	B&W	F	R frontal	Oligoastrocytoma WHO II/IV
12	CTR	Huashan	F	L parietal	Glioblastoma multiforme (WHO IV)
13	CTR	Huashan	M	R temporal	Metastases
14	CTR	Huashan	F	L posterior temporal	Oligodendroglioma WHO II

All the MRIs were resampled to a uniform image spacing 1 × 1 × 1 (in mm) along the x, y, z (axial, coronal, sagittal) image directions. For all the conducted experiments, we used linear displacement FE biomechanical models with 4-node tetrahedral elements, and the tissues (brain parenchyma, tumor) were modeled as elastic isotropic materials. Table 3 lists the parameters for the experiments. The values of the mechanical properties (E, ν) for the brain parenchyma and the tumor were obtained from Miga et al. (2001). Since we anticipate larger induced deformations by the tumor resection, the window search size and the number of adaptive iterations were set slightly larger in the PTR and CTR cases than in the BS cases (Table 3). Liu et al. (2014) offers more details about the PBNRR parameters.

**Table 3:** **The input parameters for the 14 clinical cases**.

**Parameters**	**Units**	**Value**	**Description**	**Module**	**Method**
*B*_sx_ × *B*_sy_ × *B*_sz_	Voxels	3 × 3 × 3	Block size	FS-BM	All
*W*_sx_ × *W*_sy_ × *W*_sz_	Voxels	7 × 7 × 7 (BS)	Window search size	BM	All
		9 × 9 × 9 (PTR, CTR)			
*F_s_*	–	5%	% of selected feature blocks	FS	All
δ	–	5	Mesh size	MG	APBNRR
*E_b_*	Pa	2.1 × 10^3^	Brain Young's modulus	FEMS	All
*E_t_*	Pa	2.1 × 10^4^	Tumor Young's modulus	FEMS	APBNRR
*ν_b_*	–	0.45	Brain Poisson's ratio	FEMS	All
*ν_t_*	–	0.45	Tumor Poisson's ratio	FEMS	APBNRR
λ	–	1	Trade off parameter	FEMS	All
*F_r_*	–	25%	% of rejected outlier blocks	FEMS	All
*N*_appr_	–	10	Number of approximation steps	FEMS	All
*N*_int_	–	5	Number of interpolation steps	FEMS	All
*N*_iter_	–	3 (BS)	Number of adaptive iterations	–	APBNRR
		4 (PTR, CTR)			

*BS, Brain Shift; PTR, Partial Tumor Resection; CTR, Complete Tumor Resection; FS, Feature Selection; BM, Block Matching; MG, Mesh Generation; FEMS, FEM Solver; All, PBNRR-APBNRR; x, axial; y, coronal; z, sagittal*.

### Quantitative results

For the quantitative evaluation, a publicly available implementation of the HD metric (Commandeur et al., 2011) computed the alignment errors HD_RIGID_, HD_PBNRR_, and HD_APBNRR_, after a rigid, a non-rigid (PBNRR), and the adaptive non-rigid (APBNRR) registration, respectively. The smaller the HD value, the better the alignment. The ratios HD_RIGID_/HD_APBNRR_ and HD_PBNRR_/HD_APBNRR_, (when > 1) represent the alignment improvement of the APBNRR compared to the rigid and the PBNRR registration, respectively. The higher the ratio, the greater the improvement. In all the case studies, the HD was computed between extracted point sets in the warped pre-op and the intra-op MRI. For the point extraction, we employed ITK's Canny edge detection method. For the APBNRR, the output warped pre-op MRI was used for the point extraction.

**Figure 4:**
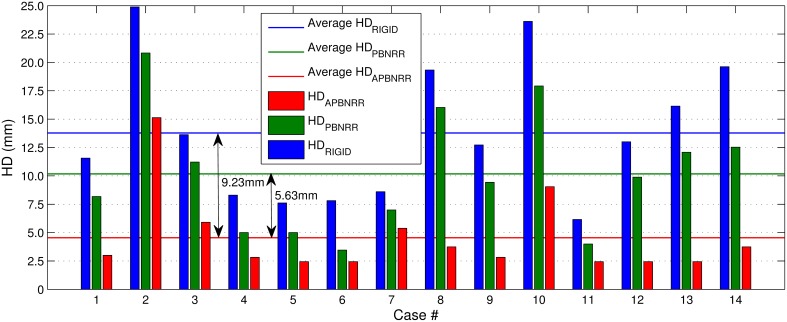
**The Hausdorff Distance (HD) alignment error for the 14 clinical cases**. The horizontal lines illustrate the average HD error of each method.

Table 4 depicts the results from the quantitative evaluation. The APBNRR registers more accurately the pre-op to the intra-op MRI, regardless of the case type (BS, PTR, or CTR). For the BS cases (1–7), our method achieves more accurate alignments, up to 3.85 and 2.72 times (in case 1), compared to the rigid registration and the PBNRR registration, respectively. Regarding the PTR and CTR cases (8–14), our method accomplishes even better results; the alignment error lowered by up to 6.61 and 4.95 times (in case 13), compared to the rigid registration and the PBNRR registration, respectively. The two-tailed *t*-Test (significant level 0.05) in Table 4 demonstrates that the alignment improvement is statistically significant for the rigid (*P*-value 2.673E-06 < 0.05) and the PBNRR methods (*P*-value 4.533E-05 < 0.05). Figure 4 depicts the HD_RIGID_, HD_PBNRR_, HD_APBNRR_, and their corresponding average values for all the experiments; the average APBNRR error is reduced by 9.23 and 5.63 mm compared to the error of other two methods.

**Table 4:** **The quantitative evaluation results for the 14 clinical cases**.

**Case**	**Type**	**HD_RIGID_**	**HD_PBNRR_**	**HD_APBNRR_**	**HD_RIGID_/HD_APBNRR_**	**HD_PBNRR_/HD_APBNRR_**
1	BS	11.57	8.18	3.00	3.85	2.72
2	BS	24.89	20.83	15.13	1.64	1.37
3	BS	13.63	11.22	5.91	2.30	1.89
4	BS	8.30	5.00	2.82	2.94	1.77
5	BS	7.61	5.00	2.44	3.11	2.05
6	BS	7.81	3.46	2.44	3.20	1.41
7	BS	8.60	7.00	5.38	1.59	1.30
8	PTR	19.33	16.03	3.74	5.16	4.28
9	PTR	12.72	9.43	2.82	4.51	3.34
10	CTR	23.61	17.92	9.05	2.60	1.99
11	CTR	6.16	4.00	2.44	2.52	1.64
12	CTR	13.00	9.89	2.44	5.32	4.05
13	CTR	16.15	12.08	2.44	6.61	4.95
14	CTR	19.62	12.53	3.74	5.24	3.35
Average		13.78	10.18	4.55	3.61	2.57
*P*-value	Two-tailed test (significance level 5E-02)				2.673E-06	4.533E-05

*HD_RIGID_, HD_PBNRR_, HD_APBNRR_ are the alignment errors after a rigid, a non-rigid (PBNRR), and an adaptive non-rigid registration (APBNRR), respectively. BS, Brain Shift; PTR, Partial Tumor Resection; CTR, Complete Tumor Resection. HD measured in millimeters*.

### Qualitative results

**Figure 5:**
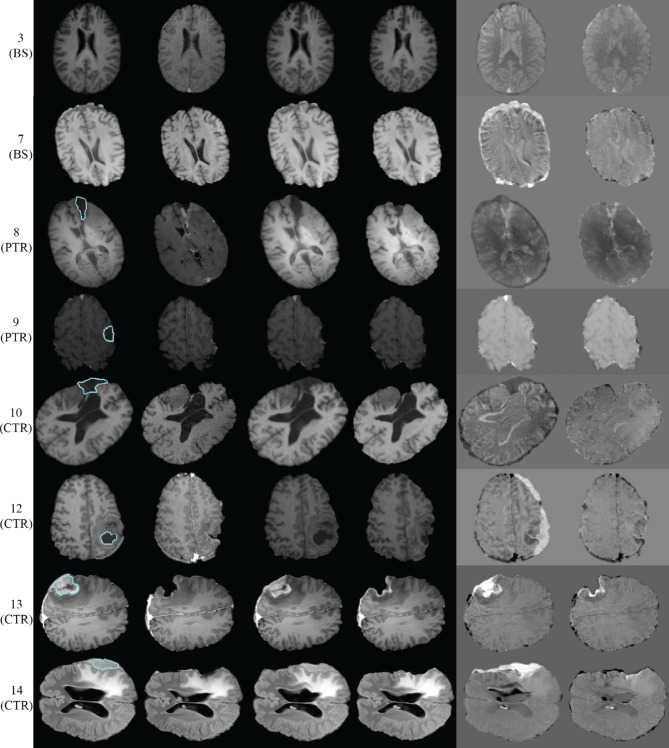
**Qualitative evaluation results for 8 of the 14 case studies**. Each row represents a single case. The **left** margin indicates the number and the type of each case. From **left** to **right** column: pre-op MRI, intra-op MRI, warped pre-op MRI (PBNRR), warped pre-op MRI (APBNRR), warped pre-op MRI (PBNRR) subtracted from intra-op MRI, warped pre-op MRI (APBNRR) subtracted from intra-op MRI. For the PTR and CTR cases, the cyan color delineates the tumor segmentation in the pre-op MRI. The BS cases do not require tumor segmentation.

Figure 5 presents the qualitative evaluation results for 8 of the 14 experiments. The BS cases 3 and 7, the PTR cases 8 and 9, and the CTR cases 10, 12, 13, and 14. We observed similar qualitative results for the rest of the cases, as well. Figure 5 shows the same representative volume MRI slice for all the images belonging to the same row. For the PTR and CTR cases, the cyan color delineates the tumor segmentation in the pre-op MRI. For the BS cases, segmentation is not required as there is no resection tumor. Also, the correction modules in Figure 1 are deactivated; however, the FE adaptivity is active. The visual comparison and inspection of PBNRR and APBNRR are based on the subtraction of the corresponding warped pre-op MRI from the intra-op MRI. The results after the subtraction (difference images) are shown in the fifth and sixth column (from the left) of Figure 5. The black and white regions in the difference images indicate larger discrepancies between the warped pre-op and the intra-op images, while the gray regions indicate smaller discrepancies. Clearly, the discrepancies in APBNRR (sixth column) are significantly smaller than those in PBNRR (fifth column), particularly near the tumor resection margins.

**Figure 6:**
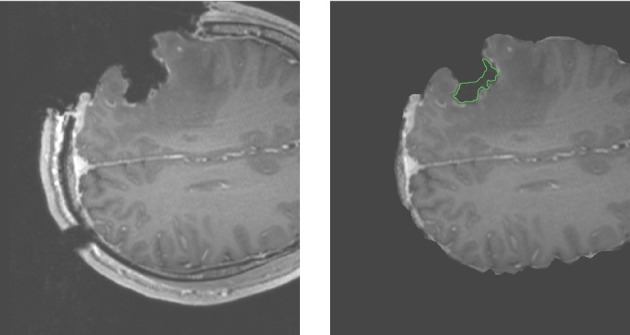
**Enhanced views of the intra-op MRI (case 13)**. The **left** figure demonstrates the brain prior to the extraction from the skull with background noise clearly visible. The **right** figure shows the brain following extraction from the skull. The background noise is mostly removed except the green demarcated region near the edges of the tumor cavity.

For case 13 of Figure 5, the APBNRR shows small misalignments at the edges of the tumor cavity, which appear like white enhancements in the corresponding difference image. These misalignments are mainly a result of remaining background noise (non-zero background intensities) in the intra-op image after pre-processing. We should point out that due to the contrast and brightness settings of Figure 5, the remaining background noise in the intra-op MRI of case 13 is not visible. In this study, for the pre-processing of the brain MRIs, we use the BET tool (Smith, 2002). Therefore, we can simultaneously remove the background noise and the skull from the brain MRIs. Figure 6 demonstrates an enhanced view of case 13; the left side depicts the original intra-op MRI (before the pre-processing) where the background noise (small white dots in the background) and the skull are clearly visible. The right side of Figure 6 exhibits the intra-op MRI that was used for the registration (after pre-processing). Though the skull is removed, a region of background noise (delineated in green) inside the tumor cavity remains. Different values for BET's fractional intensity threshold (we used the default 0.5 value) can drastically reduce or eliminate these regions and consequently avoid the local misalignments near the tumor margins.

### Performance results

All the 14 experiments were performed in a Dell Linux workstation with 2 sockets of 6 Intel Xeon X5690@3.47 GHz CPU cores each, totaling 12 cores and 96 GB of RAM. In this study, we report not only the parallel performance of our work, but also the performance of the PBNRR, as the latter consists of single-threaded components, as well as a multi-threaded component (Parallel Block Matching).

**Figure 7:**
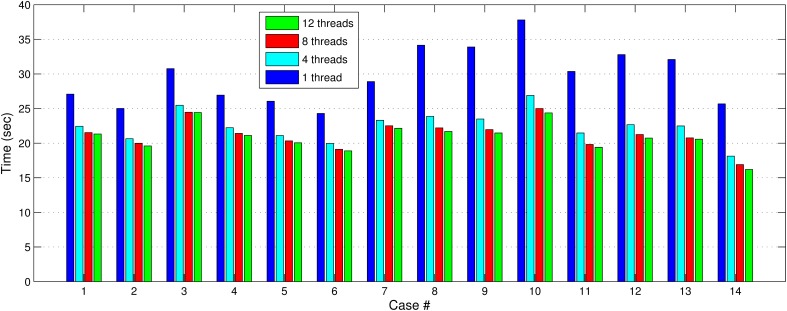
**The PBNRR total execution time for the 14 clinical cases using 1, 4, 8, and 12 hardware cores**.

**Figure 8:**
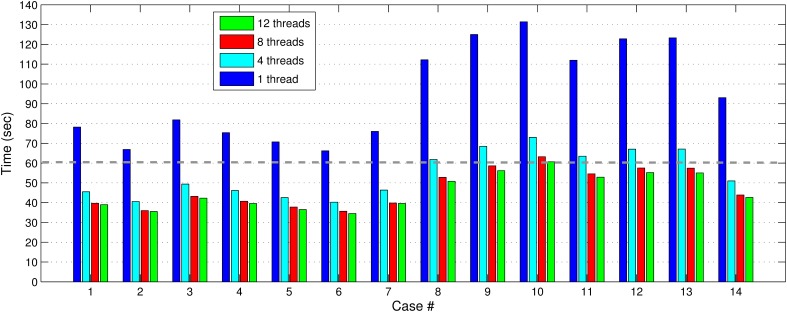
**The APBNRR total execution time for the 14 clinical cases using 1, 4, 8, and 12 hardware cores**. The gray dashed line illustrates a threshold of 60 s.

In Figures 7, 8, we report the total (end-to-end) execution times for PBNRR and APBNRR, respectively, with 1, 4, 8, and 12 threads. Apparently, our framework is computationally intensive, especially for the PTR and CTR cases where *W_s_* and *N*_iter_ are larger (Table 3).

**Figure 9:**
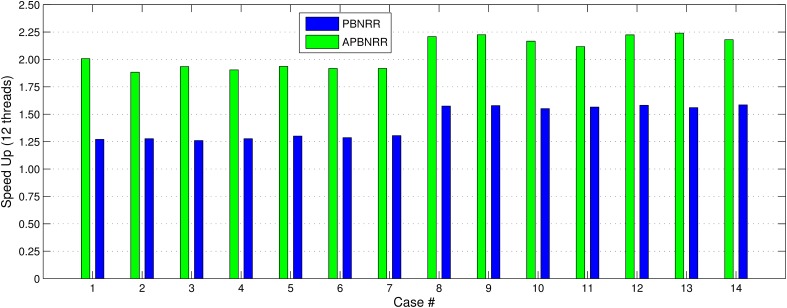
**The end-to-end speedup for the 14 clinical cases using 12 hardware cores**.

Our method exploits additional parallelism through the various implemented multi-threaded modules and achieves nearly real-time performance (between 34.51 and 60.66 s) in all the experiments, as shown with the green bars in Figure 8. Figure 9 shows the extra parallelism of our work, where the APBNRR attains greater speedup than the PBNRR in all the case studies. The reported speedup in Figure 9 corresponds to the total execution time.

Table 5 ilustrates a more detailed performance evaluation of our work; the performance for each new and existing module, the subtotal performance for the parallel modules, and the total performance for all modules (parallel + sequential), with 1 and 12 threads. The execution time with 12 threads and the speedup are not available (n/a) for the sequential modules (Warped pre-op Seg. Correction, Image Def. Field Correction and FEM Solver). According to Table 5, using 12 threads reduces the APBNRR execution time from 123.28 to 55.18 s.

**Table 5:** **The performance of the APBNRR modules for case 13 with 1 and 12 hardware cores**.

**Module status**	**Module name**	**Time (s)**		**% Total time**		**Speedup**
		**1 thread**	**12 threads**	**1 thread**	**12 threads**	
New	Parallel feature selection	15.87	4.38	12.87	7.93	3.62
	Parallel mesh generation	6.36	2.58	5.16	4.67	2.46
	Parallel JCG	5.55	3.51	4.50	6.36	1.58
	Parallel image def. field update	9.96	2.32	8.07	4.20	4.29
	Warped pre-op seg. correction	1.11	n/a	0.90	2.01	n/a
	Image def. field correction	2.62	n/a	2.12	4.74	n/a
Existing	FEM solver	33.39	n/a	27.08	60.58	n/a
	Parallel block matching	44.37	3.77	35.99	6.83	11.76
	Parallel image warping	4.05	1.50	3.28	2.71	2.70
Sub total (parallel)		86.16	18.06	69.87	32.70	4.77
Total (parallel + sequential)		123.28	55.18	100.00	100.00	2.24

In this paper, we focused on the parallelization of computational modules that all together take about 70% of the total APBNRR execution time. The remaining 30% correspond to the sequential modules. As a result, we achieved a speedup of about 4.8 for the parallel modules and about 2.25 when we consider both the parallel and the sequential modules (Table 5). The parallelization of the FEM Solver, including the assembly of the system matrices and the outlier rejection, was presented in our previous work at Liu et al. (2009). This work is not yet integrated in the APBNRR framework or the ITK system; therefore, we currently use the existing sequential ITK implementation of FEM Solver. In the future, we will incorporate the Parallel FEM Solver in our framework. The other two sequential modules (Warped pre-op Segmented Correction and Image Def. Field Correction) listed in Table 5, were not parallelized because their contribution to the total execution time is very small (0.90 and 2.12%, respectively).

## Discussion

The quantitative and qualitative performance evaluation on 14 clinical cases makes evident that the proposed method has potential use in the operating room. According to the quantitative results of Table 4, the APBNRR is up to about five times more accurate than the PBNRR method of ITKv4.5 in the 14 cases we tried, including craniotomy induced BS, partial tumor (PTR), and CTR. Figure 5 demonstrates the higher accuracy of our method, where the black and white misaligned regions in the subtracted images are significantly reduced around the resected tumor margins.

It is important to note that the large, complex deformations induced by the tumor resection deteriorate the quality of the tetrahedral elements and usually lead to elements with small dihedral angles or even flipped elements with negative volume. This is neither physically acceptable nor numerically feasible, indicating the need for choosing an adaptive FE biomechanical model to estimate the brain deformations, as is applicable to our case. We deployed, for this reason, a robust Delaunay mesh generation technique (Foteinos and Chrisochoides, 2013), which facilitates the FE model adaptivity, thereby creating high quality tetrahedral elements in parallel from a gradually warped pre-op segmented MRI. Rather than a local mesh generation, a global mesh generation is preferable because the introduced overhead is negligible in the former compared to the geometric complexity of the latter. For example, the parallel Mesh Generation module accounts for only 4.67% of the total APBNRR time with 12 running threads (Table 5). In Table 1, we demonstrate that global meshing eliminates the poor quality elements in terms of dihedral angles during the APBNRR execution.

Although the linear tetrahedron is known to be a poor element in terms of convergence (Benzley et al., 1995) in comparison to the linear hexahedron or higher order elements like the quadratic tetrahedron, we prefer it for two reasons; first, it adapts with smaller distortions to the curved or complex anatomical structures of the brain, compared to the hexahedron, so fewer elements are sufficient for an accurate representation of the brain morphology; second, the higher polynomial order elements (Bathe, 1996) introduce additional nodes on their edges, which indicates extra computational resources and time. Nevertheless, in the future we will investigate the impact of the hexahedral, higher order tetrahedral, or even hybrid meshes (Joldes et al., 2009) to test the accuracy and the performance of our method.

Regarding the performance of this study, we characterize the proposed framework as nearly real-time. The evaluation in subsection Performance Results shows that the APBNRR in all the conducted experiments can register the MRI volumes in about 1 min (i.e., between 34.51 and 60.66 s according to Figure 8). We should point out that the improvement on the APBNRR performance is limited when more than four cores are used (Figures 8, 9). This can be explained by Amdahl's Law which states that the speedup of a parallel program is limited by the time needed for the sequential fraction of the program. The maximum speedup based on Amdahl's law is given by the equation: speedup = 1/(*s* + *p/n*), where *s* and *p* is the sequential and parallel fraction of the program, respectively, and *n* is the number of the cores (threads). For our method *s* = 30.1%, *p* = 69.9% (Table 5) and the maximum total speedup obtained from the equation above with *n* = 4, 8, 12 cores is 2.10, 2.57, and 2.78, respectively. It is evident that even if the speedup of our parallel modules is linear with the number of the cores, the total APBNRR speedup is always limited to the maximum computed values from Amdahl's law. However, there is still some room for improvement. With the integration of the parallel FEM Solver (Liu et al., 2009) in our work, the parallel fraction of APBNRR will increase by approximately 97% (Table 5), the execution time is expected to decrease to less than 40 s and the maximum total speedup from Amdahl's law will be 3.66, 6.61, and 9.02 with *n* = 4, 8, and 12 cores, respectively.

## Summary and conclusion

In this study, we focus on a very challenging problem: NRR of brain MR images that compensates for brain shift and tumor resection. The ITK open-source, cross-platform system is the foundation of our framework; it implements a new feature-based NRR method that currently uses a two-tissue (brain parenchyma, tumor) patient-specific adaptive FE biomechanical model to warp the pre-op to the intra-operative MRI. The framework is capable of using any number of tissues. The registration of the volume MRIs is performed gradually, while the FE model is adaptively changing to reflect the current morphology of the brain. Currently, the new contributed modules are not integrated in ITK because their compliance with the ITK system is a cumbersome and time consuming process and is outside the scope of this project.

The proposed method is an important step toward a clinically applicable real-time NRR technology, for the following reasons:

- The quantitative and qualitative evaluation on clinical data shows promising results. The provided alignments are up to 4.95 times more accurate compared to the publicly available PBNRR method of ITKv4.5.
- The new parallel modules including the parallel Delaunay mesh generation and the parallel JCG linear solver reduce the overheads due to adaptivity and bring the end-to-end execution within the time constraints imposed by IGNS. As a result, we can register a pair of adult brain volume MRIs in about 60 s. We anticipate further improvement in the performance (about 30–50%) after future software updates.
- All software components of the framework are modularized. Thus, we enforce the maintainability and the extensibility of our scheme by the open source community and third-party developers.

### Conflict of interest statement

The authors declare that Old Dominion University filed for a provisional patent related to the research presented in this publication and thus there is a potential commercial or financial relationships that could be viewed as a potential conflict of interest if and when the patent is awarded.

## Footnotes

^1^http://www.slicer.org/

^2^http://www.itk.org/Doxygen/html/index.html
